# Investigation of Microbial Community Shifts under the Mizumoto Japanese Traditional Sake Brewing Process Using Chemical Analyses and High-throughput Sequencing

**DOI:** 10.1264/jsme2.ME23066

**Published:** 2025-06-17

**Authors:** Shinnosuke Okuhama, Yuki Nakashima, Tsumugi Nakamoto, Masataka Aoki, Yuga Hirakata, Takashi Yamaguchi, Masataka Kusube

**Affiliations:** 1 Advanced Engineering Faculty, National Institute of Technology, Wakayama College, Japan; 2 Applied Chemistry and Biochemistry, National Institute of Technology, Wakayama College, Japan; 3 Regional Environment Conservation Division, National Institute for Environmental Studies (NIES), Japan; 4 Bioproduction Research Institute, National Institute of Advanced Industrial Science and Technology (AIST), Japan; 5 Department of Science of Technology Innovation, Nagaoka University of Technology

**Keywords:** mizumoto-sake, high-throughput sequencing, Japanese sake

## Abstract

Over the past 10 centuries, sake brewing methods have been developed in stages, including doburoku, mizumoto, kimoto, yamahaimoto, and sokujyomoto. Mizumoto-sake is considered the oldest prototype. The brewing process involves lactic acid fermentation and multiple parallel saccharification and alcoholic fermentation by indigenous microbes, which has been operated based on a sense of craftsmanship. The processes involved lead to the creation of extreme conditions characterized by low pH levels and high alcohol concentrations. The characteristic feature of mizumoto-sake is that it begins with fermentation by indigenous lactic acid bacteria to produce acidic water for yeasts to ferment alcohol by inhibiting the growth of undesirable microbes. In the present study, we investigated changes in the microbial community and the transition of metabolites that affect taste and flavor during processes from the initiation of mizumoto-sake brewing to the final product. In the lactic acid fermentation phase, bacteria, including those in the genera *Lactococcus*, *Leuconostoc*, and *Lactobacillus*, produced lactic acid and contributed to the production of acidic water (pH of approximately 4) called soyashimizu. A heating process, known as “*Anka*”, which increased the brewing temperature, then switched the relative abundance of 18S rRNA from 75.0% *Pichia* to 72.3% Saccharomycetaceae. Alcohol fermentation was accelerated by the *Saccharomyces* family (relative abundance: 89.8%), reaching alcohol concentrations >15%.

Sake is a popular Japanese drink made from rice by multiple parallel fermentation, including saccharification and alcoholic fermentation. One of the original sake brews is mizumoto-sake, which was described in the Gosyuno-Nikki and Harima-Fudoki book approximately 600 years ago (Muromachi era in Japan) (Akiyama, 1978; Matsuzawa *et al.*, 2002; Kitagaki and Kitamoto, 2013). Microbes in the starter of mizumoto-sake (“*mizu*” and “*moto*” mean water and seed mash, respectively) comprise only indigenous lactic acid bacteria (LAB) and yeasts (Matsuzawa *et al.*, 2002). One of the characteristics of the mizumoto-sake process is the preparation of acidic water, known as soyashimizu, by the lactic acid fermentation of raw rice and well water. This independent process produces the specific flavor of the final product (Katagiri *et al.*, 1934; Koyanagi *et al.*, 2016; Takahashi *et al.*, 2021). Another interesting feature of mizumoto-sake is that the Toji, the sake craftsman, performs a unique operation known as the “*Anka*” treatment in shubomoromi, during which the brewing temperature is adjusted to approximately 5°C above room temperature (Fig. 1). Although there are various theories, kimoto-sake is considered to be based on mizumoto brewing with some improvement. One of the differences during the first fermentation process between mizumoto-sake and kimoto-sake is whether rice is steamed. To avoid heavy labor, yamahaimoto-sake omits the process of mashing rice and koji until they become liquid during the moromi process. Sokujyomoto-sake and some yamahaimoto-sake brewing processes prepare acidic water using chemical materials, resulting in a significant difference in taste and flavor from mizumoto-sake. Furthermore, the shubomoromi step of mizumoto-sake involves mixing soyashimizu with steamed rice and koji, and then transferring this mixture to a new vessel for sugar and alcohol fermentation for approximately two months (Fig. 1) (Nishio and Shigeru, 2008; Bokulich *et al.*, 2014; Koyanagi *et al.*, 2016). This step changes the microbial community in the brewing vessel, thereby accelerating fermentation. Mizumoto-sake is produced via 3-dan shikomi (adding ingredients and water three separate times) and the scale of moto is 15-fold higher than that of shubomoromi (Fig. 1). Based on their experience, the Toji decides the timing of the transition to the next step by considering the pH index, the smell of organic acids, and the yeast condition on the surface (Fig. 1). Mizumoto-moromi corresponds to the moromi step of other sake types. Therefore, shubomoromi and mizumoto-moromi promote alcohol fermentation, producing an alcohol concentration >15%.

Previous studies investigated the microbial community of‍ ‍kimoto-sake and/or yamahaimoto-sake and revealed that‍ ‍the dominant prokaryote species were *Lactobacillus*, *Bacillus*, *Leuconostoc*, *Tetragenococcus*, *Staphylococcus*, *Acinetobacter*, and *Klebsiella*, while the dominant eukaryote species were *Wickerhamomyces*, *Saccharomyces*, and *Aspergillus* (Matsuzawa *et al.*, 2002; Bokulich *et al.*, 2014; Koyanagi *et al.*, 2016). These species include LAB, such as *Leuconostoc mesenteroides* and *Lactobacillus sakei*, and nitrate-reducing bacteria (NRB), such as *Pseudomonas*, *Escherichia*, and *Enterobacter*. They produce several organic acids and decrease pH to inhibit spoilage due to bacteria and yeast during the initial process (Katagiri *et al.*, 1934; Kitahara *et al.*, 1957; Tsuji *et al.*, 2018; Takahashi *et al.*, 2021).

The initial process, soyashimizu, to make “*moto*” is the simplest brewing approach and is also a sustainable brewing process that naturally selects the desired microbial community for fermentation, compared to kimoto-sake and/or yamahaimoto-sake. Moreover, the decisive difference between mizumoto-sake and other sakes is the acidification of the brewing water by the high percentage of LAB. This water contains lactic acid in the initial stages of preparation, which inhibits the growth of undesirable microbes. “*Anka*”, a warming process, encourages the growth of yeast and leads to the fermentation of alcohol. In the present study, we investigated microbial community shifts, particularly during the processes of soyashimizu and shubomoromi, using high-throughput sequencing and chemical ana­lyses of the final products by microbes that affect taste and flavor to obtain a more detailed understanding of the oldest sustainable fermentation system, mizumoto-sake brewing.

## Materials and Methods

### Fermentation sample collection

**Fig. 1.**
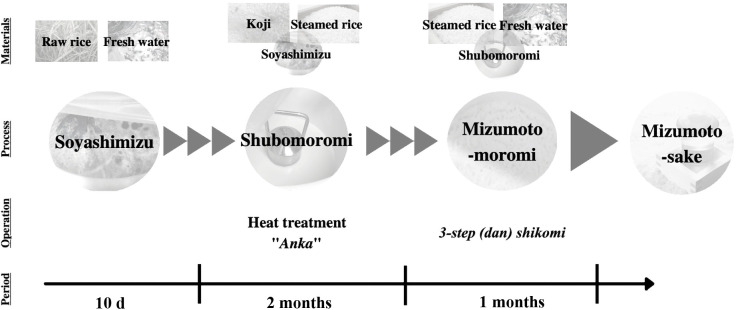
Mizumoto-sake production scheme.

Soyashimizu was prepared by soaking raw rice, purchased from a local farmer, in fresh well-water and then incubating it at room temperature for 10 days (Fig. 1). Soyashimizu samples were collected on days 1, 2, 7, and 10 after soaking. A mixture of steamed rice and koji, which consisted of *Aspergillus oryzae* incubated on steamed rice, was added to soyashimizu in order to prepare shubomoromi, also known as “moto”. The mixture was then incubated for two months. In the final step, mizumoto-moromi was brewed for one month in scaled-up casks with an approximate capacity of 2,200 L, and samples were collected after 3-dan shikomi (Fig. 1). Steamed rice was saccharified with koji and mixed into shubomoromi and mizumoto-moromi. All fermentation samples were collected and stored at –20°C until DNA extraction was performed.

### Microbial population of each fermentation stage

Fermenting microbes in each sample collected (2‍ ‍μL for bacteria and 7‍ ‍μL for yeast) were suspended by sonication at 35 kHz for 15‍ ‍min in 500‍ ‍μL distilled H_2_O. Sonicated solutions were gently fixed with 200‍ ‍μL of 3.7% formaldehyde and incubated at room temperature for 15‍ ‍min. Fixed samples were stained with SYBR GREEN I (Takara Bio) on a polycarbonate membrane filter with a pore size of 0.22‍ ‍μm (Hawach Scientific) and the cell concentration was quantified using a fluorescence microscope (Eclipse E600, Nikon). The number of bacteria mL^–1^ was estimated from a count of at least 3 randomly selected microscopic fields and a total of at least 100 bacteria were counted in all samples. An eye piece fitted with a micrometer disk was used to delineate a portion of the field for counting (Hobbie *et al.*, 1977).

### Sequencing

Total genomic DNA extraction from all samples was performed using ISOIL for the Beads Beating kit (Nippon Gene). The approximately 300-bp prokaryotic 16S rRNA gene V4 region was amplified by the ExTaq Hot Start Version (Takara Bio) using the universal primers 515F (5′-GTGCCAGCMGCCG CGGTAA-3′) and 806R (5′-GGACTACHVGGGTWTCTAAT-3′). PCR was performed by denaturation at 94°C for 5‍ ‍min, followed by 30 cycles at 94°C for 0.5‍ ‍min, 57°C for 1‍ ‍min, and 72°C for 1.5‍ ‍min and a final extension at 72°C for 7‍ ‍min. The 18S rRNA gene variable region was amplified by the ExTaq Hot Start Version (Takara Bio) using the universal primers V4-1F (5′-CCAGCASCYGCGGTAATWCC-3′) and TarR (5′-ACTTTCGTTCTTGATYRA-3′). PCR was performed as follows: denaturation at 94°C for 3‍ ‍min, followed by 15 cycles at 94°C for 0.5‍ ‍min, 53°C for 0.75‍ ‍min, and 72°C for 1‍ ‍min, followed by 25 cycles at 94°C for 0.5‍ ‍min, 48°C for 0.75‍ ‍min, and 72°C for 1‍ ‍min, and a final extension at 72°C for 10‍ ‍min (Hirakata *et al.*, 2016, 2019).

All PCR products were purified using a QIAquick PCR purification kit (Qiagen) according to the manufacturer’s instructions. The DNA concentrations of prepared amplicon libraries were quantified using Qubit dsDNA HS Kits (Thermo Fisher Scientific) and measured using Qubit 2.0 (Thermo Fisher Scientific). The quality of the sequencing ana­lysis was confirmed using a PhiX spike-in control with MiSeq (Illumina).

### Data ana­lysis

Low-quality sequence reads (quality score ≤30) from ambiguous nucleotides were excluded from the Miseq library. The amplicon sequences obtained were classified into the same operational taxonomic unit (OTU) at a sequence similarity level of 97% after the removal of chimera sequences. The taxonomical classification of each OTU representative read was performed using mothur software v1.39.5. based on SILVA database v138 (Schloss, 2009). Microbial species richness as alpha-diversity indices (Chao1 and Shannon indices) was calculated using mothur software. A principal coordinate ana­lysis (PCoA) was also calculated using the same analytical software (Koyano, 2012). PICRUSt2 (v2.4.1) was used to predict microbial functions based on the KEGG pathway database (Douglas *et al.*, 2019; Zhao *et al.*, 2021). All statistical analyses were performed using R language (http://www.R-project.org/) (R Core Team, 2019). Plots were generated using the ggplot2 package (Wickham, 2016).

### Flavor compound ana­lysis

Flavor compounds were extracted from 2‍ ‍mL of mizumoto-moromi or sokujyomoto-moromi (brewed for this study) using MonoTrap (GL Sciences). Samples were filtered through a polycarbonate membrane filter with a pore size of 0.22‍ ‍μm (Hawach Scientific). An absorbing carbon disk was then placed at the same height in each bottle and incubated at 60°C for 1 h. Absorbed volatile components were extracted in 400‍ ‍mL dichloromethane by 35‍ ‍kHz sonication for 5‍ ‍min, and analyzed by Agilent 7890A Gas Chromatograph (Agilent Technologies) with flame ionization detection on JMS-QI050GC (JEOL) and a DB-WAX UI GC column (30 m, 0.25‍ ‍mm, 0.25‍ ‍μm) (Agilent Technologies). The initial oven temperature was 40°C for 10‍ ‍min, followed by an increase to 200°C at a rate of 10°C min^–1^, and then held for 2‍ ‍min.

### Organic acid compounds

One milliliter of each sample was filtered through a polycarbonate membrane filter with a pore size of 0.22‍ ‍μm (Hawach Scientific) after centrifugation at 9,170×*g* (KUBOTA3740) at room temperature for 1‍ ‍min for the HPLC ana­lysis. The HPLC Prominence system (Shimadzu) comprised a post-column pH-buffered electric conductivity detector, Shim-pack SCR-102H column (300‍ ‍mm: length [L]×8.0‍ ‍mm: inner diameter [i.d.]), guard column SCR-102H (50‍ ‍mm L×6.0‍ ‍mm i.d.), and system controller CBM-20A. Injected solutions (10‍ ‍μL) were separated at 40°C at a flow rate of 0.8‍ ‍mL‍ ‍min^–1^ using 5‍ ‍mmol L^–1^ *p*-toluenesulfonic acid as the mobile phase. The sample was detected at 43°C at a flow rate of 0.8‍ ‍mL‍ ‍min^–1^ using 5‍ ‍mmol L^–1^ *p*-toluenesulfonic acid, 20‍ ‍mmol L^–1^ of Bis-Tris, and 0.1‍ ‍mmol L^–1^ EDTA∙4H_2_O as the post-column reaction buffer.

### Fermentation conditions of mizumoto-sake

pH was measured using a pH meter of the Navi D-50 series (Horiba). Fermented ethanol concentrations were assessed using the Alcomate AL-3 apparatus (RIKEN KEIKI). The Brix index, an indicator of sugar concentrations, was evaluated using the refractometer ASRE1000 (AS ONE) after filtering samples through a polycarbonate membrane filter with a pore size of 0.22‍ ‍μm (Hawach Scientific).

### Nucleotide sequence accession numbers

The prokaryotic and eukaryotic nucleotide sequences reported in the present study were deposited in the DDBJ/EMBL/NCBI GenBank databases with the following accession numbers: SRR17185524–SRR17185535 and SRR17184730–SRR17184745, respectively.

## Results

### General results of the metagenomic ana­lysis

**Fig. 2.**
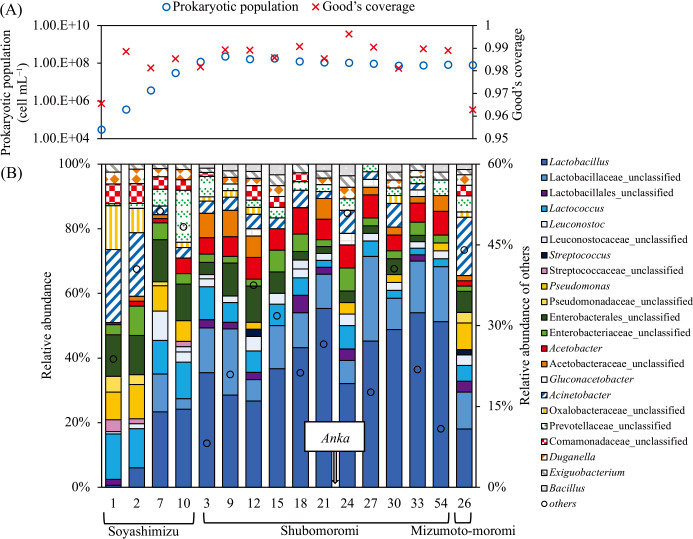
Prokaryotic community by the V4 region of the 16S rRNA gene. The numbers on the horizontal axis represent the sampling time (day). (A) Prokaryotic population and Good’s coverage, and (B) prokaryotic flora community using MiSeq (only taxa detected at <1% OTU maximum relative abundance are shown).

**Fig. 3.**
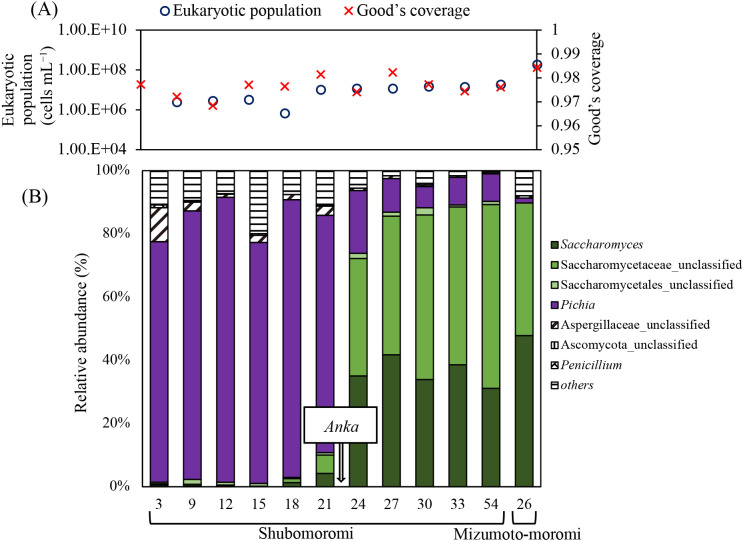
Eukaryotic community by the V4 region of the 18S rRNA gene. The numbers on the horizontal axis represent the sampling time (day). (A) Eukaryotic population and Good’s coverage, and (B) eukaryotic flora community using MiSeq (only taxa detected at <0.1% maximum relative abundance are shown).

Total reads from the high-throughput sequencing ana­lysis were 108,319 and 209,277 for the V4 regions of the prokaryotic 16S rRNA and eukaryotic 18S rRNA genes, respectively. Total prokaryotic and eukaryotic OTUs were 1,374 and 5,078, respectively, as assessed by mothur v1.35.1 software. Good’s coverage from raw data yielded values of 0.963–0.996 and 0.968–0.984, as shown in Fig. 2A and 3A, respectively. The alpha-diversity indices Chao1 and Abundance-based Coverage Estimator (ace) for species richness and Simpson and Shannon for evenness were calculated (Table S1). The highest Chao1 and ace indices for prokaryotic and eukaryotic richness were observed on day 1 in soyashimizu and day 9 in shubomoromi, respectively. The lowest Chao1 and ace indices for prokaryotic richness were noted on day 24 in shubomoromi. The lowest Chao1 and ace indices for eukaryotic richness were obtained on day 27 in shubomoromi and day 26 in mizumoto-moromi, respectively. High species richness was observed in the early stages of each process, and decreased over time. The Shannon index showed the highest prokaryotic and eukaryotic evenness on day 7 in soyashimizu and on day 24 in shubomoromi, respectively, while the lowest prokaryotic and eukaryotic evenness was noted on day 24 in shubomoromi and on day 26 in mizumoto-moromi, respectively. The increase in the Shannon index of 18S rRNA during shubomoromi indicated the greater diversity of eukaryotic species. These results suggest that prokaryotic species were abundant in soyashimizu samples, while the abundance of eukaryotic species increased after the shubomoromi transition.

### Microbial community and organic acid shifts in soyashimizu fermentation

**Fig. 4.**
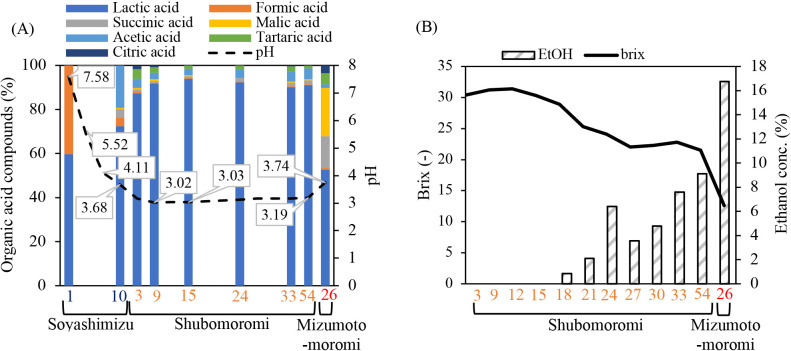
Results of chemical ana­lyses. (A) Organic acids and pH, and (B) ethanol concentrations and the Brix index. The numbers on the horizontal axis represent the sampling time (day).

**Fig. 5.**
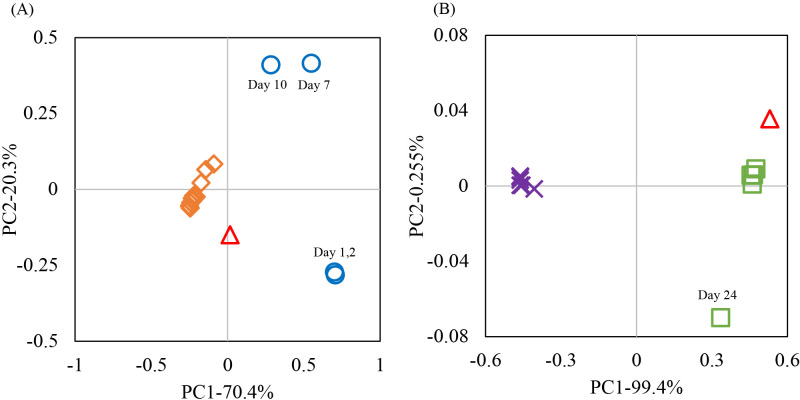
Beta-biodiversity of (A) 16S rRNA and (B) 18S rRNA according to PCoA parameters. Bubbles indicate alpha-biodiversity by the Shannon index. (A: Soyashimizu [circle], Shubomoromi [rhombus] and Mizumoto-moromi [triangle], B: 3-21 days in Shubomoromi [cross], 24–54 days in Shubomoromi [square] and Mizumoto-moromi [triangle])

We identified dominant LAB, such as *Lactococcus*, *Leuconostoc*, and *Lactobacillus*, during mizumoto-sake brewing after the detection of NRB and acetic acid bacteria (Fig. 2B). On day 1 in soyashimizu, the relative abundance of total putative LAB was 21.4%, which included *Lactobacillus* (0.6%), *Lactobacillaceae_*unclassified (0.6%), *Lactobacillales_*unclassified (1.8%), *Lactococcus* (14.1%), *Leuconostoc* (0.6%), and *Streptococcaceae*_unclassified (3.7%) (Fig. 2B). NRB were at 29.4% relative abundance, including *Enterobacterales*_unclassified (12.9%), followed by *Pseudomonas* (8.6%), *Pseudomonadaceae*_unclassified (4.9%), and *Enterobacteriaceae*_unclassified (3.1%) (Fig. 2B). The only putative acetic acid producer detected was *Acetobacter* (0.6% relative abundance) on day 1 in soyashimizu, which was similar to that reported for kimoto-sake (Kersters *et al.*, 2006; Sakurai *et al.*, 2011). The relative abundance of NRB was subsequently maintained at approximately 20–30% in soyashimizu, while *Pseudomonadaceae*_unclassified was not detected on day 10 in soyashimizu (Fig. 2B). The relative abundance of cocci LAB slightly decreased from 18.4% (day 1) to 17.7% (day 10), while that of switched bacilli LAB increased from 2.45% (day 1) to 27.4% (day 10) in soyashimizu (Fig. 2B). The prokaryotic population simultaneously shifted from 3.0×10^4^ (day 1) to 3.0×10^7^ (day 10) cells mL^–1^ and pH decreased because of the production of several acids in soyashimizu (Fig. 2 and 4A). PICRUSt2 putatively indicated that functional genes with the highest concentration were aldehyde dehydrogenase (EC 1.2.1.3), tartrate dehydrogenase (E.C 1.1.1.93), and fumarate hydrolase (E.C 4.2.1.2), which are involved in the production of acetic acid, tartaric acid, and formic acid, respectively (Fig. S1) (Smidt *et al.*, 2008; Ling *et al.*, 2020). The indigenous microbes present on day 10 in soyashimizu produced organic acids, including lactic acid, acetic acid, and formic acid, with the measured percentages of these compounds being 72.4, 19.2, and 4.1%, respectively. The shift in their composition may have been affected by the prokaryotic community composition within the brewing vessel (Fig. 4A). PCoA plots on days 1 and 2 in soyashimizu were similar under the effects of the dominant NRB and acetic acid bacteria (Fig. 5A). However, the PCoA index for the final period of soyashimizu significantly changed from that for the initial counterpart. Furthermore, the prokaryotic population density increased exponentially from 3.0×10^4^‍ ‍cells‍ ‍mL^–1^ on day 1 to 3.0×10^7^‍ ‍cells‍ ‍mL^–1^ on day 10 in soyashimizu (Fig. 2A).

### Microbial community diversity and metabolized organic acids after shubomoromi

In shubomoromi, the dominant prokaryotic community switched to *Lactobacillus* (relative abundance: 35.4%) and *Lactococcus* (relative abundance: 10.1%) in a new fermentation vessel (Fig. 2B). This transition led to decreases in alpha-biodiversity indices (Table S1). The prokaryotic cell number simultaneously increased to 1.2×10^8^‍ ‍cells‍ ‍mL^–1^, maintaining pH at approximately 3 due to the production of lactic acid (87.3%) and several other organic acids on day 3‍ ‍in shubomoromi (Fig. 2A and 4A). Towards the final brewing stage in shubomoromi, beta-biodiversity indices converged (Fig. 5A) due to the dominance of *Lactobacillus* in the brewing vessel (Fig. 2B). Regarding the eukaryotic community, the two main genera, *Pichia* and *Saccharomyces*, were detected in this step. On day 3 in shubomoromi, the genus *Pichia* accounted for 76.0% relative abundance, while Aspergillaceae, which was present in koji, accounted for 10.8% (Fig. 3B). During days 21 to 24 in shubomoromi, a shift in dominant species from *Pichia* to *Saccharomyces* occurred, maintaining the eukaryotic population at between 1.1×10^7^ and 1.2×10^7^‍ ‍cells‍ ‍mL^–1^ (Fig. 3A). This microbial community shift was attributed to the “*Anka*” treatment (incubation at room temperature+approximately 5°C between days 21 and 24 in shubomoromi), which is performed by the Toji. Beta-biodiversity indices varied depending on the dominant microbes in the brewing vessel (Fig. 5B). After the “*Anka*” treatment, the relative abundance of Saccharomycetaceae was >70%, while the Shannon and ace indices of prokaryotes gradually decreased (Table S1). On day 54 in shubomoromi, *Saccharomyces* fermented glucose into approximately 9.1% ethanol (Fig. 4B). On day 24 in shubomoromi, several genes, including alcohol dehydrogenase (E.C 1.1.1.1) and lactate dehydrogenase (E.C 1.1.1.27), which are enzymes in the TCA cycle, exhibited the highest concentrations (Fig. S1) (Smidt *et al.*, 2008; Ling *et al.*, 2020). Organic acids in mizumoto-moromi, including lactic acid (52.6%), malic acid (21.9%), and succinic acid (14.5%), which affect the taste of the product, were produced (Fig. 4A).

### Microbial community shift and final fragrance components of mizumoto-sake

**Fig. 6.**
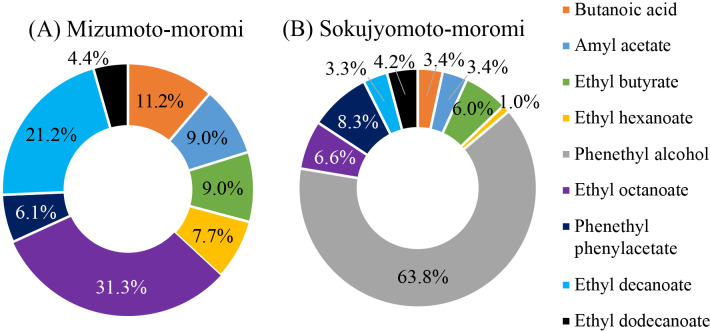
Flavor compounds in the moromi step. (A) Mizumoto-moromi, (B) sokujyomoto-moromi (K7; Kyokai yeast No. 7)

In the final step of mizumoto-moromi, the prokaryotic community slightly shifted to that in soyashimizu due to 3-dan shikomi, in which fresh raw materials were added three times. For example, the relative abundance of *Lactobacillus*, which was 24.4% on day 10 in soyashimizu, decreased from 51.2% (day 54 in shubomoromi) to 18% (mizumoto-moromi) (Fig. 2B). Mizumoto-moromi affected the taste of mizumoto-sake. We detected characteristic flavor components, such as ethyl octanoate (composition: 31.3%), ethyl decanoate (composition: 21.2%), butanoic acid (composition: 11.2%), amyl acetate (composition: 9.0%), ethyl butyrate (composition: 9.0%), and ethyl hexanoate (composition: 7.7%) (Fig. 6A). We compared the ratio of flavor components in mizumoto-moromi to those in sokujyomoto-moromi, which is the most common procedure for sake brewing. Phenethyl alcohol, the base of sake flavor, was detected in sokujyomoto-moromi (63.8%), but not in mizumoto-moromi (Fig. 6). The ratios of amyl acetate, ethyl butyrate, and ethyl hexanoate were higher in mizumoto-moromi (9.0, 9.0, and 7.7%, respectively) than in sokujyomoto-moromi (3.4, 6.0, and 1.0%, respectively) (Fig. 6). The relative abundance of sake yeast, including *Saccharomyces* and Saccharomycetaceae, was 89.8%; therefore, the concentration of ethanol reached 16.5% (Fig. 3B and 4B). The microbial community was maintained under specific conditions during brewing, such as a high ethanol concentration (16.5%) and low pH (3.7).

## Discussion

Mizumoto-sake has been brewed by multiple parallel fermentation processes using an indigenous microbial community. Soyashimizu, the first and most essential fermentation step to promote acidification, is the main factor responsible for the quality of the final product and eliminates microbes that are not suitable for brewing (Fig. 1). Sake brewing typically starts during the cold season in Japan, between December and March, because brewing may be initiated at a low temperature (approximately 0°C) to inhibit the growth of unexpected microbes. In contrast, mizumoto brewing starts by soaking raw rice in well water at approximately 15°C in October to establish acidic conditions using indigenous microbes.

### Preparation of acidic conditions in primary fermentation for mizumoto-sake

During the initial process called soyashimizu in mizumoto-sake, NRB, such as *Enterobacteriaceae* and *Pseudomonas*, contribute to the reduction of nitrate to nitrite. *Enterobacteriaceae* are characterized by their ability to ferment glucose and produce several organic acids and carbon dioxide. Therefore, these NRB not only provide an acidic environment, but also induce the pKa shift in organic acids, which is essential for the growth of LAB and wild yeast to become the dominant species (Rehr and Klemme, 1989; Sato *et al.*, 2016). However, in kimoto-sake, the *Pseudomonas* population, not *Enterobacteriaceae*, is selected as the dominant community member during the initial fermentation period (Rehr and Klemme, 1989; Nishio and Shigeru, 2008; Sato *et al.*, 2016). Several LAB, including *Lactococcus*, *Leuconostoc*, *Lactobacillus*, and *Streptococcus*, have been discovered in both kimoto-sake and mizumoto-sake (Momose and Kamao, 1993; Masuda *et al.*, 2012; Bokulich *et al.*, 2014; Koyanagi *et al.*, 2016; Wang *et al.*, 2018). *Tetragenococcus*, which is common in yamahaimoto-sake, was not detected in mizumoto-sake (Apriyantono, 1996; Koyanagi *et al.*, 2016).

The difference during the first fermentation process between kimoto-sake and mizumoto-sake is whether rice is steamed. The pH value on day 7 in soyashimizu (4.1) was similar to that on day 7 in kimoto-sake (3.8) (Bokulich *et al.*, 2014; Koyanagi *et al.*, 2016). This result suggests that the starter solution is acidified by using either raw or steamed rice as ingredients, providing a suitable environment for sake brewing. Several kimoto-sake and yamahaimoto-sake brewing processes involve artificial preparations for favorable conditions, either by inoculations with LAB/yeast or the addition of chemical compounds (Katagiri *et al.*, 1934; Masuda *et al.*, 2012; Sato *et al.*, 2016; Tsuji *et al.*, 2018).

**Fig. 7.**
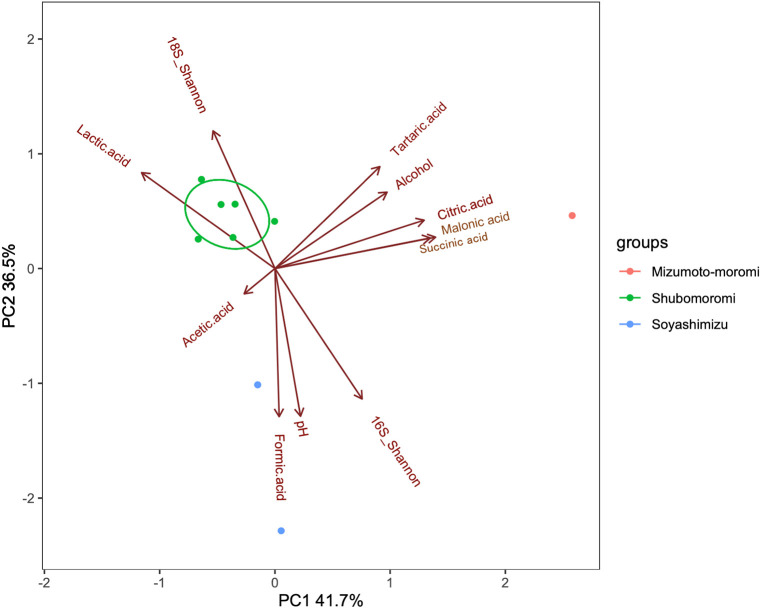
Principal component ana­lysis of alpha-biodiversity and chemical components. 16S_Shannon: alpha-biodiversity of prokaryotes, 18S_Shannon: alpha-biodiversity of eukaryotes.

Soyashimizu, composed of raw materials and indigenous bacteria, may affect the first fermentation process as follows. Fig. 7 shows that soyashimizu samples correlate with formic acid, pH, and alpha biodiversity of prokaryotes. *Bacillus* spp. were detected in soyashimizu (Fig. 2B), and may be involved in the amylolytic process because they are commonly detected in Japanese sake and contribute to saccharification (Taman *et al.*, 2016). Furthermore, *Duganella* spp., which are present on rice plants, were detected in the initial fermentation vessel. This strain has been reported to produce antibacterial products, such as siderophores and violacein, which inhibit the growth of pathogens around plant roots (Aranda *et al.*, 2011). *Bacillus* were detected from day 3 in shubomoromi, and may be involved in the amylolytic process because they are commonly detected in Japanese sake and contribute to saccharification (Taman *et al.*, 2016). *Prevotella* belong to *Prevotellaceae*, found in mizumoto-sake, and synthesize short-chain fatty acids from carbohydrates, such as polysaccharides and xylose (Chen *et al.*, 2017).

### “*Anka*” operation to convert microbe diversity

“*Anka*”, an operation performed by the Toji, which controls the fermentation temperature in the vessel at approximately 5°C above room temperature from days 21 and 24 in shubomoromi, affected the microbial community and induced it to start vigorous ethanol fermentation. Even when there are no measuring instruments, the Toji identifies changes in the microbiota through keen observations and makes appropriate judgments.

*Pichia*, a film yeast, is the nitrite producer that maintains the LAB population (Passoth *et al.*, 2006; Shimaoka *et al.*, 2012), and was the dominant genus on day 3 in shubomoromi (Fig. 3B). It formed a film on the water surface of the tank and the population ratio decreased after the “*Anka*” treatment (Fig. 3B). Conversely, *Saccharomyces* (relative abundance: 35.1%) and Saccharomycetaceae (relative abundance: 37.2%) increased over *Pichia* (relative abundance: 19.9%) on day 24 in shubomoromi after the “*Anka*” treatment (Fig. 3). We considered the “*Anka*” treatment to have changed the brewing flora from *Pichia* to *Saccharomyces* and resulted in alcohol accumulation due to the high abundance of alcohol dehydrogenase derived from *Saccharomyces* (Smidt *et al.*, 2008; Ling *et al.*, 2020). To control the yeast population and enhance the concentration of ethanol, the “*Anka*” treatment, which originated in mizumoto-sake, has become a common practice of other traditional brewing processes, such as kimoto-sake and yamahaimoto-sake (Akada, 2002; Kanauchi, 2013).

In the final period of shubomoromi, a relative abundance of 89.8% for *Saccharomyces* and Saccharomycetaceae was detected, and the concentration of ethanol reached 9.1% (Fig. 3B and 4B). Therefore, the warm “*Anka*” treatment prompted alcohol fermentation through the growth of indigenous *Saccharomyces* (Fig. 3).

### Microbial community shift and final fragrance components of mizumoto-sake

Several specific fragrance components were detected in mizumoto-moromi that were not present in sokujyomoto-moromi, which is currently a common brewing procedure. Various organisms, particularly *Enterobacteriaceae*, may produce fatty acids and organic acids that contribute to the fragrance of sake (Martens *et al.*, 1992; Nishio and Shigeru, 2008).

During the maintenance of high ethanol concentrations (>10%) and low pH (<3.7), the prokaryotic population exhibited a slight decline. The microbial groups selected under these extreme conditions appear to be essential for brewing. The Toji sterilizes the microbiota through a heat treatment known as “*Hiire*”, which complies with the Food Sanitation Law.

By employing high-throughput sequencing and general chemical ana­lyses, we detected a shift in the microbial community in each stage during mizumoto-sake fermentation, and observed the participation of the indigenous microbial community. A more detailed understanding of the microbial fermentation process that occurs in an unsterile environment using indigenous microbes will provide insights into the history and brewing process of sake. The microbial community ana­lyses performed in this study allow for the scientific visualization of parallel fermentation in Japan 600 years ago.

## Citation

Okuhama, S., Nakashima, Y., Nakamoto, T., Aoki, M., Hirakata, Y., Yamaguchi, T., and Kusube, M. (2025) Investigation of Microbial Community Shifts under the Mizumoto Japanese Traditional Sake Brewing Process Using Chemical Analyses and High-throughput Sequencing. *Microbes Environ ***40**: ME23066.

https://doi.org/10.1264/jsme2.ME23066

## Supplementary Material

Supplementary Material
