# Supplementary material for: Investigation of Microbial Community Shifts under the Mizumoto Japanese Traditional Sake Brewing Process Using Chemical Analyses and High-throughput Sequencing
Source: Microbes Environ. 2025 Jun 17;40(2):ME23066. doi: 10.1264/jsme2.ME23066 (PMC12213057; doi:10.1264/jsme2.ME23066)
Supplement: Supplementary file 1 — Supplementary Material [file 40_23066_s1.pdf]

Fig. S1 Shinnosuke Okuhama

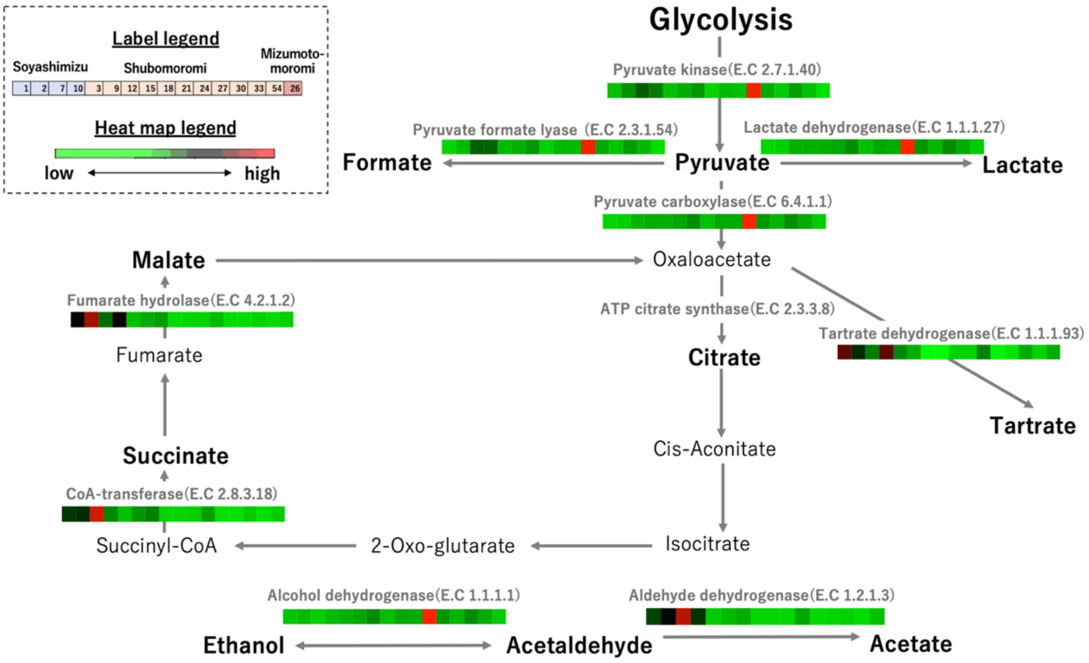

Fig. S1 Predicted functional profile of the microbial communities in mizumoto-sake using PICRUSt2

Table S1 Shinnosuke Okuhama

Table S1 Alpha bio-diversity indices during brewing period calculated by mothur

Sake Sample		Chao1		ace		Shannon		Simpson	
Period [days]		16S rRNA	18S rRNA	16S rRNA	18S rRNA	16S rRNA	18S rRNA	16S rRNA	18S rRNA
Soyashimizu	1	302.7	-	650.9	-	2.459	-	0.1518	-
	2	101.0	-	149.6	-	2.201	-	0.1702	-
	7	167.8	-	380.7	-	2.525	-	0.1095	-
	10	153.8	-	301.6	-	2.322	-	0.1436	-
Shubomoromi	3	135.7	1569	218.1	4815	1.246	0.4425	0.5272	0.8919
	9	88.49	2747	129.2	11108	1.092	0.3930	0.5844	0.9133
	12	80.39	1785	142.5	4767	0.9218	0.4955	0.6613	0.8889
	15	120.3	1700	220.1	6097	0.7454	0.4491	0.7447	0.8795
	18	82.44	1468	183.1	4441	0.5464	0.3801	0.8202	0.9146
	21	127.7	1012	244.5	3286	0.6321	0.5086	0.7954	0.8332
	24	25.14	1416	33.24	4065	0.2149	0.8521	0.9352	0.6254
	27	87.74	921.5	161.1	2688	0.2891	0.5166	0.9157	0.8071
	30	133.5	1441	265.3	5725	0.5833	0.5529	0.8298	0.8117
	33	114.1	1998	230.8	5197	0.4988	0.5507	0.8379	0.8216
	54	125.8	1608	201.8	4841	0.3941	0.5724	0.8788	0.8023
Mizumoto-moromi	26	256.4	1210	546.2	1765	1.981	0.2466	0.3198	0.9448
